# Spectrum of Non-Nucleoside Reverse Transcriptase Inhibitor-Associated Drug Resistance Mutations in Persons Living with HIV-1 Receiving Rilpivirine

**DOI:** 10.3390/v16111715

**Published:** 2024-10-31

**Authors:** Pavithra Nagarajan, Jinru Zhou, Giulia Di Teodoro, Francesca Incardona, Carole Seguin-Devaux, Rolf Kaiser, Ana B. Abecasis, Perpetua Gomes, Kaiming Tao, Maurizio Zazzi, Robert W. Shafer

**Affiliations:** 1 Division of Infectious Diseases, Department of Medicine, Stanford University, Stanford, CA 94305, USAkaiming.tao@stanford.edu (K.T.); 2 EuResist Network GEIE, 00152 Rome, Italyf.incardona@informa.pro (F.I.); 3 Department of Information Engineering, University of Pisa, 56122 Pisa, Italy; 4 InformaPRO S.r.l., 00152 Rome, Italy; 5 Department of Infection and Immunity, Luxembourg Institute of Health, L-4354 Esch-sur-Alzette, Luxembourg; 6 Institute of Virology, University of Cologne, 50931 Cologne, Germany; 7 Deutsches Zentrum fuer Infektionsforschung, 38124 Braunschweig, Germany; 8 Global Health and Tropical Medicine, LA-REAL, Instituto de Higiene e Medicina Tropical, Universidade NOVA de Lisboa, 1349-008 Lisbon, Portugal; 9 Laboratório de Biologia Molecular, Serviço de Patologia Clínica, Unidade Local de Saúde Lisboa Ocidental, Hospital Egas Moniz, 1349-019 Lisbon, Portugal; 10 Egas Moniz Center for Interdisciplinary Research (CiiEM), Egas Moniz School of Health & Science, Caparica, 2829-511 Almada, Portugal; 11 Department of Medical Biotechnology, University of Siena, 53100 Siena, Italy

**Keywords:** rilpivirine, HIV-1 drug resistance, non-nucleoside reverse transcriptase inhibitors

## Abstract

Introduction: Few data are currently available on the nonnucleoside reverse transcriptase (RT) inhibitors (NNRTI) resistance mutations selected in persons living with HIV-1 (PLWH) who develop virological failure while receiving rilpivirine (RPV). Methods: We analyzed pooled HIV-1 RT genotypic data from 280 PLWH in the multicenter EuResist database and 115 PLWH in the Stanford HIV Drug Resistance Database (HIVDB) who received RPV as their only NNRTI. Results: Among the 395 PLWH receiving RPV, 180 (45.6%) had one or more NNRTI-associated DRMs. Overall, 44 NNRTI-associated DRMs were identified, including 26 that occurred in two or more PLWHs. Seven mutations had a prevalence ≥10% among the 180 PLWH with one or more NNRTI-associated DRM: E138K (32.2%), V90I (25.0%), K101E (17.8%), Y181C (17.2%), E138A (13.9%), H221Y (12.2%), and K103N (10.6%). Y181C was significantly more likely to co-occur with K101E, V179F, H221Y, and M230L. Ten novel non-polymorphic mutations at known NNRTI-associated mutation positions were also identified, usually in just one PLWH: L100F, V108A, T139I, P225S, M230V, Y232C, and T240A/I/M/S. Conclusions: Our analysis extends the spectrum of mutations emerging in PLWH receiving RPV. Additional phenotypic characterization of RPV-selected mutations is necessary to better understand their biological and possible clinical significance.

## 1. Introduction

A regimen containing a long-acting injectable nanosuspension of rilpivirine (RPV-LA) in combination with a long-acting formulation of the integrase strand transfer inhibitor (INSTI) cabotegravir (CAB-LA) was recently approved as a bi-monthly treatment for patients with stable virological suppression for six or more months on antiretroviral therapy (ART). This regimen, henceforth CAB/RPV, has proven to be highly effective with a confirmed virological failure (VF) rate of about 1% in phase III clinical trials, including ATLAS, FLAIR, and ATLAS-2M [1,2]. While this risk of VF is quite low, VF in persons living with HIV (PLWH) receiving CAB/RPV is of concern because it has been associated with emergent resistance to both RPV and CAB, and cross-resistance to the other second-generation INSTIs, dolutegravir, and bictegravir. Moreover, because RPV is well-tolerated, it has been commonly used to simplify therapy in PLWH who have attained prolonged virological suppression on less well-tolerated regimens [3,4]. Recent post hoc multivariable analysis has suggested that the presence of two or more of the following baseline factors were associated with an increased risk of VF on CAB/RPV, including baseline RPV resistance-associated mutations, A6 subtype, and a body mass index ≥ 30 kg/m^2^ [5,6].

Our current knowledge of RPV-resistance mutations is limited. As of September 2024, the Stanford HIV Drug Resistance Database (HIVDB) contained data on 131 isolates from 115 previously NNRTI-naïve persons, most of whom had VF on a first-line RPV-containing regimen [7,8,9]. Moreover, HIV-1 RT sequences (as opposed to lists of amino acid mutations) were available for only 69 of these 131 isolates. Therefore, we decided to expand our knowledge about potential RPV-resistance mutations by pooling data from HIVDB with sequences from previously NNRTI-naïve PLWH with VF on an RPV-containing regimen from the large multicenter EuResist Integrated Database (EIDB) [10].

## 2. Methods

We analyzed HIV-1 group M RT amino acid mutations obtained from previously NNRTI-naïve PLWH receiving RPV. Sequences were obtained from two sources: (1) HIVDB [11] and (2) the multicenter EIDB [10]. HIVDB contained previously published nucleotide sequences and lists of amino acid mutations. The EIDB contained complete nucleotide sequences that had not been previously published. Ethical approval was obtained in the respective host countries of the original databases that contributed data to the EIDB.

All sequences were submitted to the HIVDB genotypic resistance interpretation program to extract the following information: (1) the positions in RT that were sequenced; (2) HIV-1 subtype; (3) the number of stop codons, deletion mutations, and signature APOBEC mutations; (4) the mutations assigned a mutation penalty score for any NNRTI; and (5) non-polymorphic mutations that did not receive a mutation penalty score but have been previously reported to be significantly more common in NNRTI-experienced compared with NNRTI-naïve persons, which we refer to as unscored non-polymorphic NNRTI-selected mutations [12].

NNRTI-associated drug-resistance mutations (DRMs) were defined as mutations meeting one of the following criteria: (1) received an HIVDB mutation penalty score for one or more NNRTIs; (2) unscored non-polymorphic NNRTI-selected mutations [12]; and (3) mutations reported in the previously published weighted genotype score for resistance to etravirine (ETR), an NNRTI that is structurally similar to RPV [13].

We performed three analyses using the pooled data from HIVDB and the EIDB. First, we characterized the prevalence of each NNRTI-associated DRM in this combined dataset. We excluded NNRTI-associated DRMs that were known to be present prior to the administration of the RPV-containing regimen. When more than one sample was available from the same person, we defined the mutation list as the union of all mutations present in the samples from that person.

Second, we examined the frequency with which NNRTI-associated DRMs co-occurred in the same virus isolates using pairwise Spearman correlation coefficients, restricting our analysis to mutations that occurred three or more times in the dataset.

Third, to identify potentially novel RPV-selected mutations, we analyzed those mutations that were not defined as NNRTI-associated DRMs. For this analysis, we compared the prevalence of these mutations to their prevalence in sequences from NRTI-experienced NNRTI-naïve persons in HIVDB. This group was selected as a control because it was expected that some uncommon NRTI-selected mutations would be enriched among the sequences from RPV-treated persons.

To determine the distance between RPV and those positions with potential novel RPV-associated mutations, we used the *2zd1* 1.8 Å resolution crystal structure of HIV-1 RT in complex with RPV [14].

## 3. Results

HIVDB contained RT genotype sequences (n = 69) or lists of mutations (n = 62) for 131 isolates from 115 PLWH who received an RPV-containing regimen. Of the 131 isolates, most were from two clinical trials and one clinical cohort [7,8,9]. Notably, only mutation lists were available from the largest of these studies [7]. The EIDB contained 320 RT sequences from 280 previously NNRTI-naïve PLWH who received an RPV-containing regimen. Table 1 summarizes the demographics, subtypes, and treatment histories of the combined 395 PLWH.

All sequences encompassed RT 1-230; 51.9% encompassed RT 1-300. Samples were derived from plasma in 77.0%, peripheral blood mononuclear cells (PBMCs) in 16.2%, and whole blood in 4.1%. The source was not known for 2.7% of samples. The most common HIV-1 subtypes included subtype B (61.5%), subtype A (9.4%), CRF02_AG (6.8%), subtype C (6.6%), and subtype G (5.8%). Sequences from 21 PLWH were excluded because they contained ≥2 signature APOBEC mutations, ≥1 stop codon, or ≥1 deletion mutation. Of the 14 sequences with ≥2 signature APOBEC mutations, nine were from PBMCs, three from whole blood, one from plasma, and one from an unspecified source.

Of the 115 PLWH in HIVDB, 80 (69.6%) had one or more NNRTI-associated DRM. Of the 259 PLWH in the EIDB with sequences meeting quality control criteria, 100 (38.6%) had one or more NNRTI-associated DRM. Table 2 shows the prevalence of each NNRTI-associated DRM in the pooled dataset, its prevalence in ART-naïve persons, and its RPV HIVDB mutation penalty score as of September 2024. For mutations that did not receive a mutation penalty score, the table indicates which mutations were previously reported to be non-polymorphic and significantly associated with NNRTI exposure [12] and which mutations were included in the weighted ETR genotype resistance score [13].

Overall, 44 NNRTI-associated DRMs at 18 RT positions occurred in one or more PLWH (Table 2). Seven mutations had a prevalence of ≥10% including V90I, K101E, K103N, E138K/A, Y181C, and H221Y. E138K was the most common RPV-selected mutation occurring in 59 PLWH–32.2% of those with an NNRTI-associated mutation. It occurred with the NRTI-resistance mutation M184I in 35 persons, with the NRTI-resistance mutation M184V in 20 persons, and with 21 different NNRTI-associated mutations, of which the most common were V90I (n = 11), H221Y (n = 9), and K101E (n = 6). Of note, there were no other statistically significant correlations between NRTI and NNRTI-associated DRMs after adjustment for multiple comparisons.

Among the 180 PLWH with an NNRTI-resistance DRM for whom ART status prior to receiving an RPV-containing regimen was known, E138K was significantly more likely to occur among the 72 PLWH known to be ART-naïve than among the 84 PLWH known to be ART-experienced (51.4% vs. 17.9%; *p* = 0.00001; Fisher’s Exact Test). In contrast, K103N was more likely to occur among those known to be ART-experienced (16.7% vs. 2.8%; *p* = 0.006; Fisher’s Exact Test).

Among the 180 PLWH with an NNRTI-resistance DRM, E138K occurred more commonly among the 147 PLWH who received tenofovir disoproxil fumarate or tenofovir alafenamide in combination with emtricitabine plus RPV than among the 33 who received a different regimen (35.4% vs. 18.2%; *p* = 0.06; Fisher’s Exact Test). K101E was significantly more likely to occur among the 119 subtype B isolates compared to the 61 non-B isolates (22.7% vs. 8.2%; *p* = 0.02; Fisher’s Exact Test). E138A was more likely to occur in the non-B isolates (19.7% vs. 10.9%; *p* = 0.1; Fisher’s Exact Test). The presence of virological suppression prior to receiving an RPV-containing regimen did not influence the spectrum of NNRTI-associated DRMs. As we did not have access to the plasma HIV-1 RNA level at the time of virus sequencing, we were unable to determine whether there was an association between the specific NNRTI-associated mutations that were selected and the virus load.

Twenty-eight of the 44 NNRTI-associated DRMs in the dataset had a non-zero RPV mutation penalty score in the HIVDB genotypic resistance interpretation program: A98G, L100I/V, K101E/H/P, V106I, E138A/G/K/Q/R, V179D/E/F/L, Y181C/I, Y188F/L, G190A/E/Q/S, H221Y, F227C, and M230I/L. Ten of the mutations had a mutation penalty score of zero for RPV: K103N/S, V106A, V108I, Y188H, P225H, F227L, L234I, and K238N/T. Six mutations did not receive a mutation penalty score for any NNRTI, including four unscored non-polymorphic NNRTI-selected mutations—K101N/T, V179T, and F227Y—and two mutations in the ETR weighted genotypic score (V90I and V179T).

Table 3 shows those pairs of RPV-selected NNRTI-associated DRMs that co-occurred in three or more PLWH and were positively correlated with one another and those pairs of negatively correlated NNRTI-associated DRMs. Four pairs of mutations had an absolute Spearman rho value ≥0.2 and an uncorrected *p*-value < 0.01. The strongest positive correlations involved Y181C in combination with K101E, V179F, H221Y, and M230L. E138K was negatively correlated with both K103N and Y181C.

Table 4 lists 11 potentially novel mutations at NNRTI-associated DRM positions that had a prevalence ≥3-fold higher in RPV-treated persons compared with NRTI-experienced NNRTI-naïve persons: L100F (n = 1), V108A (n = 1), T139I (n = 2), I178V (n = 14), P225S (n = 1), M230V (n = 1), Y232C (n = 1), and T240A/I/M/S (n = 6). I178V is a polymorphic mutation with a prevalence of 1.2% in NRTI-experienced NNRTI-naïve persons. The remaining mutations had a prevalence of <0.1% in this control population. L100F occurred with E138A. T139I occurred with E138K. P225S occurred with T240S. T240A occurred with Y188L and E138G in one sample and with E138A in another sample. T240I occurred with K101E, Y181C, and G190A while T240I/M occurred with K101H/N and K103N. V108A, M230V, and Y232C did not occur with any other NNRTI-associated DRMs.

Table 5 shows the distance from the closest atom in the wild-type residue to the closest atom in RPV at each of the 22 positions with one or more NNRTI-associated DRM in the X-ray structure *2zd1.* Nearly all of the positions (n = 19) were within eight Angstroms. For two positions, 138 and 139, the closest residue was in the p51 chain. Among the potential novel RPV-associated mutations, positions 139, 178, 232, and 240 were generally further away from RPV (range: 6.8 to 9.8 Angstroms) than the predefined NNRTI-associated DRM positions (2.7 to 11.0 Angstroms).

## 4. Discussion

RPV is a second-generation NNRTI that was approved in 2011 as an oral drug and in 2022 in a long-acting injectable form in combination with CAB. Its efficacy results in part from its structural flexibility in conformations with the flexible hydrophobic NNRTI binding pocket [14]. However, at its approved dosage, RPV has a relatively low genetic barrier to resistance. Indeed, based on initial clinical trials, its recommended use was restricted to patients with a plasma HIV-1 RNA level ≤10^5^ copies per/mL [16]. It is, therefore, tempting to speculate that mutations minimally reducing RPV susceptibility may influence the success of an RPV-containing ART regimen.

The mutations identified in this study in a person receiving RPV overlapped considerably with mutations that have previously been reported to emerge during in vitro passage experiments with RPV [17,18,19,20,21,22]. Indeed, in 46 independent passage experiments, the 10 most commonly selected DRMs were E138K (28 experiments), L100I (16 experiments), Y181C (14 experiments), M230I (10 experiments), K101E (10 experiments), F227C (8 experiments), V108I (7 experiments), M230L (6 experiments), V106A (4 experiments), and H221Y (3 experiments). Six of these 10 mutations—E138K, Y181C, K101E, H221Y, V108I, and L100I—were among the 10 most frequently occurring mutations in persons receiving RPV. The remaining four of the most frequently selected mutations in vitro, M230L, F227C, V106A, and M230I, occurred in 5.6%, 2.2%, 1.7%, and 0.6% of persons receiving RPV. Conversely, three of the most prevalent mutations in persons receiving RPV—E138A, K103N, and V106I—were not selected in vitro, while V90I, the second most prevalent mutation in persons receiving RPV, was selected in just one passage experiment.

Of the 17 mutations at nine positions on the IAS-USA list of RPV-associated resistance mutations [23], 16 were selected among the PLWH in our dataset: K101E, E138A/K, Y181C, and H221Y occurred in ≥10% of persons receiving RPV; L100I, M230L, and Y181I in 5% to 10%; K101P, E138G/Q, Y188L, and F227C in 1% to 5%; and E138R, V179L and M230I in <1%. The IAS-USA RPV-associated resistance mutation Y181V was not present in our dataset.

Our data confirm that E138K was more likely to occur in combination with M184I (n = 35) rather than M184V (n = 20), which usually occurs much more frequently than M184I. This phenomenon was initially observed in the ECHO, THRIVE, and STaR clinical trials [7,9]. Biochemical, cell culture and structural studies have suggested that the combination of E138K and M184I (rather than M184V) is associated with increased replication in the presence of ETR or RPV and a cytosine analog [24,25,26,27]. Additionally, the combination of E138K and Y181C has been shown to reduce RT processivity in biochemical assays, likely explaining the negative that we observed between these two mutations [28].

Considering the large size of the dataset analyzed in this study, it is likely that some NNRTI-associated DRMs, particularly those that occurred less frequently, may not have been selected by RPV but rather may have been present prior to the use of RPV as a result of transmitted drug resistance. One of the limitations of this study is that baseline genotypes were available for only 117 (29.6%) persons. Indeed, among those persons who had genotypic resistance tests before receiving an RPV-containing regimen, seven (6.0%) had K103N/S. Although these seven mutations were removed from our analysis, it is likely that additional mutations, particularly at position 103, may have also been transmitted. K103N has been the most commonly transmitted drug-resistance mutation in many studies. It was detected in 8.6% of previously untreated persons in the U.S. between 2014 and 2018 [29], while K103N/S was reported in 9.1% of persons in a large recent European study [30]. However, we believe that the combination of K103N plus L100I, which occurred in three PLWHs in this study, was likely selected by RPV, as this combination has been reported to occur in ART-naïve persons receiving RPV in a clinical trial and as the combination has been shown to synergistically reduce RPV susceptibility [9,17,31].

Several NNRTI-associated DRMs that occurred commonly in our cohort did not receive HIVDB mutation penalty scores for RPV. These included eight DRMs that occurred in ≥1.0% of persons, including V90I (25.0%), K103N (10.6%), V108I (9.4%), V106A (1.7%), P225H (1.1%), F227L (1.1%), K238T (1.1%), and K238N (1.1%). A review of published phenotypic susceptibility data in HIVDB indicates that isolates containing each of these DRMs alone consistently had a <2.0-fold fold reduction in susceptibility to RPV and/or ETR. Moreover, the contribution of each of these mutations to reduced RPV susceptibility in linear regression models was minimal [32,33]. Indeed, with the exception of V90I, which had an effect similar to H221Y, each of these DRMs had an effect on RPV susceptibility less than that of the 17 IAS-USA RPV-associated DRMs. Thus, the strong selection pressure in favor of V90I and V108I in PLWH receiving RPV suggests that these DRMs may contribute to reduced RPV susceptibility or increased virological fitness only when they occur with other NNRTI-associated DRMs.

This study also identified several potential novel RPV-associated DRMs. First, four unscored non-polymorphic NNRTI-selected mutations K101N/T, V179M, and F227Y each occurred in one person receiving RPV. Second, 10 non-polymorphic mutations at a known NNRTI-associated mutation position each occurred in one-to-two persons, including L100F, V108A, T139I, P225S, M230V, Y232C, and T240A/I/M/S. Of note, T240I, which occurred in two persons, had previously been reported to be selected in vitro in two studies [17,34]. However, we could not identify published phenotypic data on isolates with these mutations. Given the increasing use of CAB/RPV, it has become particularly important to obtain more data on the phenotypic effects of RPV-selected mutations, including the potentially novel ones identified in this study.

## Figures and Tables

**Table 1:** Demographic, Clinical, and Virological Characteristics of Previously NNRTI-Naïve PLWH Receiving an RPV-Containing Regimen.

	EIDB (n = 280)	HIVDB (n = 115)	Total (n = 395)
***Age* ^1^**			
Median	43	NA	NA
Range	18–71	NA	NA
***% Male*** **^1^**			
	61%	NA	NA
***Countries*** **^2^**			
	Italy (45.0%)	United States (46.1%)	Italy (31.9%)
	Portugal (15.7%)	Unknown (46.0%)	United States (13.4%)
	Germany (14.3%)	Russia (3.5%)	Unknown (13.4%)
	Sweden (9.7%)	Netherlands (3.5%)	Portugal (11.1%)
	Luxembourg (6.4%)	Germany (0.9%)	Germany (10.4%)
	Poland (4.6%)		Sweden (6.8%)
	Russia (4.3%)		Luxembourg (4.6%)
			Poland (3.3%)
			Russia (4.1%)
			Netherlands (1.0%)
***Year of the sample***			
Median	2017	2011	2016
Range	2011–2023	2009–2023	2009–2023
***Subtypes***			
B	53.9%	80.0%	61.5%
A	11.8%	3.5%	9.4%
CRF02_AG	8.9%	1.7%	6.8%
C	7.1%	5.2%	6.6%
G	8.2%	0.0%	5.8%
Other	10.1%	9.6%	9.9%
***Source of the samples***			
Plasma	67.5%	100.0%	77.0%
PBMC	22.9%		16.2%
Whole Blood	5.7%		4.1%
Unknown	3.9%		2.7%
***ART-Naïve before RPV-containing regimen***			
Yes	14.3%	73.9%	19.7%
No	62.1%	24.3%	49.9%
Unknown	23.6%	1.8%	30.4%
***Genotypic resistance tests available prior to RPV-containing regimen***			
Yes	22.5%	47.0%	29.6%
No/Unknown	77.5%	53.0%	70.4%
***Virologically suppressed before RPV-containing regimen***			
Yes	23.6%	10.4%	31.7%
No	41.1%	71.3%	51.1%
Unknown	35.3%	18.3%	17.2%
***RPV-containing regimen***			
	TDF/FTC/RPV (60.0%)	TDF/FTC/RPV (83.5%)	TDF/FTC/RPV (66.8%)
	TAF/FTC/RPV (19.3%)	CAB/RPV (7.0%)	TAF/FTC/RPV (13.7%)
	DTG/RPV (4.3%)	DTG/RPV (4.3%)	DTG/RPV (4.3%)
	ABC/3TC/RPV (3.2%)	Other ARVs/RPV (5.2%)	ABC/3TC/RPV (2.3%)
	3TC/RPV (1.8%)		CAB/RPV (2.0%)
	Other ARVs/RPV (11.4%)		3TC/RPV (1.3%)
			Other ARVs/RPV (9.6%)

Footnote: ^1^ Age and gender were not available for the PLWH in HIVDB. ^2^ The countries were not known for the samples in the ECHO, THRIVE, and SWORD trials [7,15]. Abbreviations: TDF: Tenofovir Disoproxil Fumarate, TAF: Tenofovir Alafenamide, FTC: Emtricitabine, CAB: Cabotegravir, DTG: Dolutegravir, ABC: Abacavir, 3TC: Lamivudine, RPV: Rilpivirine. Other ARVs/RPVs generally represent more complex regimens used for late salvage therapy.

**Table 2:** Prevalence of NNRTI-Associated DRMs among PLWH receiving RPV as their only NNRTI.

Mutation	Prevalence in 180 PLWH with ≥1 NNRTI-Associated DRM	Prevalence in ART-Naïve PLWH in HIVDB ^1^	HIVDB RPV Mutation Penalty Score ^2^
E138K	32.2%	0.1%	45
V90I	25.0%	1.6%	**
K101E	17.8%	0.3%	45
Y181C	17.2%	0.4%	45
E138A	13.9%	2.3%	15
H221Y	12.2%	0.2%	15
K103N	10.6%	1.8%	0
V108I	9.4%	0.3%	0
V106I	7.8%	2.1%	5
L100I	7.2%	0.1%	60
M230L	5.6%	0.03%	60
Y181I	5.0%	0.02%	60
E138Q	4.4%	0.07%	15
K101P	4.4%	0.03%	60
A98G	3.9%	0.3%	15
Y188L	2.8%	0.1%	60
E138G	2.8%	0.3%	15
F227C	2.2%	0.004%	45
V179D	2.2%	1.6%	10
V179F	1.7%	0.01%	15
V106A	1.7%	0.005%	0
V179E	1.7%	1.2%	10
P225H	1.1%	0.1%	0
K238T	1.1%	0.1%	0
F227L	1.1%	0.04%	0
K238N	1.1%	0.06%	0
K103S	0.6%	0.08%	0
K101T	0.6%	0.01%	*
L234I	0.6%	0.01%	0
M230I	0.6%	0.01%	30
Y188H	0.6%	0.02%	0
V179L	0.6%	0.009%	15
G190Q	0.6%	0.002%	45
G190S	0.6%	0.04%	15
E138R	0.6%	0.004%	15
Y188F	0.6%	0.01%	30
F227Y	0.6%	0.03%	*
G190A	0.6%	0.4%	15
V179T	0.6%	0.5%	**
V179M	0.6%	0.01%	*
K101H	0.6%	0.03%	10
K101N	0.6%	0.01%	*
L100V	0.6%	0.01%	15
G190E	0.6%	0.02%	60

Footnote: ^1^ The total number of ART-Naïve PLWH in HIVDB was approximately 140,000 depending on the position. ^2^ According to the Stanford HIVDB mutation penalty scoring system, a penalty of 15–29 indicates low-level resistance, a penalty of 30–59 indicates intermediate resistance, and a penalty of ≥60 indicates high-level resistance. A score of 0 indicates a mutation associated with resistance to an NNRTI other than RPV. * indicates mutations that do not have a penalty score but which were previously reported to be non-polymorphic mutations selected by NNRTIs [12]. ** indicates mutations that do not have a penalty score but which were included in the weighted etravirine resistance score [13].

**Table 3:** Positive and Negative Correlations Among RPV-Selected NNRTI-Associated DRMs.

Mutation A	Mutation B	A and B	A Only	B Only	Neither A Nor B	Rho	*p* ^1^
*Positive Correlations* ^1^							
V179F	Y181C	3	0	28	149	0.29	0.0001
H221Y	Y181C	10	12	21	137	0.28	0.0001
M230L	Y181C	6	4	25	145	0.28	0.0002
K101E	Y181C	12	19	19	130	0.26	0.0005
*Negative Correlations* ^1^							
E138K	Y181C	2	55	29	94	−0.25	0.0007
E138K	K103N	1	56	18	105	−0.20	0.008

Footnote: ^1^ Spearman correlation analysis was restricted to NNRTI-associated DRMs that occurred in three or more PLWHs receiving RPV.

**Table 4:** Novel mutations at NNRTI-associated DRM positions defined as having a prevalence ≥ three-fold higher among PLWHs receiving RPV compared with previously published NRTI-experienced, NNRTI-naïve PLWHs.

Mutation	Prevalence Among 374 RPV Recipients ^1^	Prevalence in 7705 NRTI-Experienced, NNRTI-Naïve Persons in HIVDB	Prevalence Ratio (RPV/NRTI-Experienced, NNRTI-Naïve)	*p*
L100F	0.3%	0.01%	20.6	0.09
V108A	0.3%	0.01%	18.8	0.1
T139I	0.8%	0.09%	9.4	0.009
I178V	3.5%	1.2%	3.0	0.0009
P225S	0.3%	0.0%	inf	0.05
M230V	0.3%	0.0%	inf	0.05
Y232C	0.3%	0.02%	16.7	0.1
T240I	0.5%	0.0%	inf	0.004
T240M	0.3%	0.0%	inf	0.06
T240S	0.3%	0.02%	15.8	0.1
T240A	0.5%	0.08%	6.4	0.06

Footnote: ^1^ The 21 persons with sequences that did not meet the quality control criteria were removed from this analysis. Of note, even mutations that occurred just one or two times among PLWHs receiving RPV (e.g., prevalence of 0.3% or 0.5%) nonetheless often occurred ≥3 times more often compared with NRTI-experienced NNRTI-naïve persons.

**Table 5:** Distance between Rilpivirine and the NNRTI-associated mutation positions in this study in the x-ray crystal structure 2zd1 ^1^.

Pos ^2^	WT	Mutations	Distance (Angstroms)	RT Atom	RPV Atom	Chain
101	K	E, H, N, T, P	2.7	O	N4	A
234	L	I	3.3	O	N5	A
181	Y	C, I	3.4	CD2	C2	A
188	Y	F, H, L	3.5	CE2	C22	A
227	F	C, L, Y	3.5	CE1	N5	A
100	L	V, I, F	3.5	CB	N4	A
138	E	A, G, K, Q, R	3.6	OE1	C9	B
103	K	N, S	3.6	CG	C15	A
225	P	H, S	3.7	CB	N5	A
179	V	D, E, F, I, M, T	3.8	CG2	C8	A
106	V	A, I	4.0	CG2	C13	A
190	G	A, E, Q, S	4.4	N	C8	A
238	K	N, T	6.0	O	C14	A
230	M	I, L, V	6.5	N	N6	A
108	V	A, I	6.8	CG2	N6	A
139	T	I	6.8	CG2	C10	B
232	Y	C	7.3	O	N6	A
178	I	V	7.5	C	C10	A
98	A	G	8.6	C	N2	A
221	H	Y	9.2	CE1	N6	A
240	T	A, I, M, S	9.8	N	N5	A
90	V	I	11.0	O	C7	A

Footnote: ^1^ Distance from the closest atom in the wild-type residue to the closest atom in RPV. ^2^ Positions 139, 178, 232, and 240 were sites at which unscored non-polymorphic NNRTI-selected were observed in this dataset [12].

## Data Availability

Previously unpublished nucleotide sequences are available at GenBank PQ368914-PQ369225. Access to data from the EuResist Network is subject to certain restrictions. They can be requested by submitting a study application form available at https://www.euresist.org/become-a-partner (last accessed 28 October 2024), pending approval from the EuResist Network.
